# Genome-wide analysis of transmembrane 9 superfamily genes in wheat (*Triticum aestivum*) and their expression in the roots under nitrogen limitation and *Bacillus amyloliquefaciens* PDR1 treatment conditions

**DOI:** 10.3389/fpls.2023.1324974

**Published:** 2024-01-08

**Authors:** Fei Li, Kuanling Xi, Yuke Li, Tang Ming, Yufeng Huang, Lijun Zhang

**Affiliations:** ^1^ The Key Laboratory of Biodiversity Conservation in Karst Mountain Area of Southwest of China, Forestry Ministry, School of Life Sciences, Guizhou Normal University, Guiyang, China; ^2^ Science and Technology Division, Guizhou Normal University, Guiyang, China

**Keywords:** transmembrane 9 superfamily, gene family, wheat, nitrogen limitation, *Bacillus amyloliquefaciens* PDR1

## Abstract

**Introduction:**

Transmembrane 9 superfamily (TM9SF) proteins play significant roles in plant physiology. However, these proteins are poorly characterized in wheat (*Triticum aestivum*). The present study aimed at the genome-wide analysis of putative wheat TM9SF (TraesTM9SF) proteins and their potential involvement in response to nitrogen limitation and *Bacillus amyloliquefaciens* PDR1 treatments.

**Methods:**

*TraesTM9SF* genes were retrieved from the wheat genome, and their physiochemical properties, alignment, phylogenetic, motif structure, cis-regulatory element, synteny, protein-protein interaction (PPI), and transcription factor (TF) prediction analyses were performed. Transcriptome sequencing and quantitative real-time polymerase reaction (qRT-PCR) were performed to detect gene expression in roots under single or combined treatments with nitrogen limitation and *B. amyloliquefaciens* PDR1.

**Results and discussion:**

Forty-seven *TraesTM9SF* genes were identified in the wheat genome, highlighting the significance of these genes in wheat. *TraesTM9SF* genes were absent on some wheat chromosomes and were unevenly distributed on the other chromosomes, indicating that potential regulatory functions and evolutionary events may have shaped the TraesTM9SF gene family. Fifty-four cis-regulatory elements, including light-response, hormone response, biotic/abiotic stress, and development cis-regulatory elements, were present in the *TraesTM9SF* promoter regions. No duplication of *TraesTM9SF* genes in the wheat genome was recorded, and 177 TFs were predicted to target the 47 *TraesTM9SF* genes in a complex regulatory network. These findings offer valued data for predicting the putative functions of uncharacterized TM9SF genes. Moreover, transcriptome analysis and validation by qRT-PCR indicated that the TraesTM9SF genes are expressed in the root system of wheat and are potentially involved in the response of this plant to single or combined treatments with nitrogen limitation and B. amyloliquefaciens PDR1, suggesting their functional roles in plant growth, development, and stress responses.

**Conclusion:**

These findings may be vital in further investigation of the function and biological applications of TM9SF genes in wheat.

## 1 Introduction

Wheat (*T. aestivum*) is an important food crop consumed worldwide (Ismagul et al., 2014). Diverse factors, including drought, salinity, nutrient limitation, pest attack, and extreme temperatures, can affect the growth, development, quality, and yield of wheat and other crops (Zhang et al., 2021; Ahlawat et al., 2022; Dissanayake et al., 2022; Fang et al., 2022a; Venzhik et al., 2022; Wang et al., 2022; Yue et al., 2022; Yu et al., 2022). It is essential to examine the mechanisms involved in wheat growth under such conditions to improve productivity; it is also vital to boost wheat growth under these conditions. Recently, there has been much interest in exploring wheat transcriptome to elucidate how it responds to different types of stress. For instance, scientists have studied various abiotic stresses to find genes and transcription factors that could aid wheat in tolerating stress (Saidi et al., 2023). The transcriptome of wheat roots in response to drought has also been studied, pinpointing genes that could help drought tolerance (Xi et al., 2023). In addition, there have been some exciting developments in wheat omics (Tyrka et al., 2021; Sehgal et al., 2023; Szeliga et al., 2023), meaning there is much room for discoveries and research in wheat breeding. Studies have also demonstrated the presence of numerous gene families in the genetic material of wheat plants (Limin et al., 1997; Li et al., 2008; Ma et al., 2017; Peng and Yang, 2017; Qiao et al., 2017). However, despite some of these gene families being characterized and their functions having been identified, it is essential to investigate additional gene families to understand better how they are involved in wheat growth, development, or adaptation to unfavorable conditions.

Transmembrane 9 superfamily (TM9SF) proteins, also known as Nonaspanins or the endomembrane protein-70 (EMP70) family, belong to a family of proteins containing nine putative transmembrane domains and a sizeable non-cytoplasmic domain. The transmembrane domain is called the EMP70 domain. During evolution, a highly conserved group of TM9SFs is distributed in various eukaryotic species such as *Drosophila*, *Dictyostelium*, and *Saccharomyces cerevisiae*, mammals, and plants (Pruvot et al., 2010). The latest research findings suggest that when essential nutrients are deficient, TM9SFs are vital in regulating the activities of endosomes and lysosomes and are instrumental in facilitating the phagocytosis of bacteria and cellular adhesion and controlling various specific cellular functions (Pruvot et al., 2010; Perrin et al., 2015). However, despite the advances in genomic studies, TM9SFs in plants, especially wheat species, and their functional roles still need to be well studied. Identifying and elucidating the evolution and role of putative TM9SF proteins in wheat may be vital for improving agricultural properties and applications of wheat.

Studies have demonstrated that wheat cultivation is closely associated with bacterial communities that play crucial roles in its growth and immunity against diseases (Carril et al., 2021; Wang et al., 2021; Youseif et al., 2021). Among these bacterial communities, plant-growth-promoting bacteria (PGPB) are of utmost importance for various plant functions such as phosphorus (P) solubilization, phytohormone production, nitrogen (N) fixation, and siderophore synthesis. The interactions among the wheat and numerous PGPB species have been reported in previous studies, and the application of PGPB in enabling and increasing sustainable wheat biomass yields under diverse conditions such as nitrogen limitation, drought, and salinity are well documented (Smyth et al., 2011; Di Benedetto et al., 2017; Di Benedetto et al., 2019; Vannini et al., 2021). Thus, PGPB and wheat interaction may be a good tool for studying the interaction of wheat with *rhizobacteria*. Studies indicated that PGPB could mobilize organic and mineral-bound nutrients and are essential players in nitrogen fixation in plants; nitrogen-fixing bacteria have been reported to be involved in the improvement of root growth, reduction of N losses from agricultural ecosystems and increase of plant resilience to environmental stresses in wheat (Kant et al., 2010; Dal Cortivo et al., 2017; Di Benedetto et al., 2017; Maenhout et al., 2018; Wang et al., 2020). Our recent study reported that *B. amyloliquefaciens* PDR1 interacts with *Arabidopsis thaliana* and provides beneficial effects for this plant (Li et al., 2020). Specifically, *B. amyloliquefaciens* PDR1 enhanced the resistance of *A. thaliana* to alkaline stress by modulating the activity of plasma membrane H+-ATPase in the roots. However, to our knowledge, studies have yet to be reported on the effect of *B. amyloliquefaciens* PDR1 on gene regulation in wheat. In addition, no previous study has reported the involvement of the *TM9SF* genes in the molecular mechanisms of a PGPB effect on wheat growth, especially under nitrogen limitation conditions. Exploring this aspect may help improve practical PGPB applications in wheat cultivation.

Thus, in the present study, we identified and performed a genome-wide analysis of putative *TM9SF* genes via comprehensive phylogenetic, chromosomal location, gene structure, and expression change analyses under single or combined treatments with nitrogen limitation and *B. amyloliquefaciens* PDR1. The results of the present work offer essential data for additional functional characterization of various genes in wheat.

## 2 Materials and methods

### 2.1 Identification of *TraesTM9SF* genes in wheat

For the identification and characterization of the *TraesTM9SF* genes in the *T. aestivum* genome, wheat CDS, proteins, gff3, and genome data were downloaded from the wheat genome database (International Wheat Genome Sequencing Consortium (IWGSC) at https://www.wheatgenome.org/). The known TM9SF protein sequences of *A. thaliana* were retrieved and used to perform BLASTp search against wheat proteins at an E-value cutoff of 10^−10^. The retrieved putative TM9SF protein sequences were further confirmed by BLASTp against Hidden Markov Model of TM9SF (PF02990) downloaded from the protein families (Pfam) database (http://pfam.xfam.org/). We downloaded putative TM9SF protein sequences of *A. thaliana* (AthTM9SFs) from The Arabidopsis Information Resource (TAIR) database (https://www.arabidopsis.org/), while the putative TM9SF protein sequences of *Sorghum bicolor* (*SbiTM9SFs*), *Setaria italica* (*SiTM9SFs*), and *Oryza sativa* (*OsTM9SFs*) were retrieved from the corresponding genomes (accession number GCF_000003195.3 for *S. bicolor*, GCF_000263155.2 for *S. italica*, and GCF_001433935.1 for *O. sativa*) downloaded from the NCBI Genome Database (https://www.ncbi.nlm.nih.gov/genome/). The conserved domains within these putative TM9SF protein sequences were retrieved by the search against the Pfam V33.1-18271 PSSMs in the NCBI Conserved domains (https://www.ncbi.nlm.nih.gov/Structure/cdd/wrpsb.cgi) to check for the presence of EMP70 domain (PF02990) before subsequent analyses.

### 2.2 Physiochemical properties, alignment, and phylogenetic analyses of putative *TraesTM9SF* genes

The physicochemical properties of the *TraesTM9SF* genes, including the protein length, molecular weight (M.W), isoelectric point (pl), aliphatic index (Ai), instability index (II), theoretical net charge and Boman (Potential Protein Interaction) index were computed using the R package Peptides (Osorio et al., 2015). The subcellular localization of *TraesTM9SF* genes was analyzed using the CELLO online program (http://cello.life.nctu.edu.tw/, (Yu et al., 2006)). The amino acid composition of the protein sequences of *TraesTM9SF* genes was calculated using the Molecular MEGA7 program (Kumar et al., 2016) and visualized as a chord plot using the chordDiagram function from the circlize package in R (Gu et al., 2014). The phylogenetic analyses were achieved using the MEGA7 program to align full-length protein sequences of *TraesTM9SFs, AtTM9SFs, SbTM9SFs, SiTM9SFs, and OsTM9SFs*. After alignment, the sequences were used for neighbor-joining (NJ) tree construction with MEGA software with default settings, except for the bootstrap, which was adjusted to 1,000 replicates. The phylogenetic tree was visualized in MEGA7. The distribution of *TraesTM9SF* genes on wheat chromosomes was retrieved from the wheat genomic and annotation files using the TBtools software (Chen et al., 2020). The Sequence Identity and Similarity (SIAS) domain (http://imed.med.ucm.es/Tools/sias.html) was used for pairwise identity and similarity analysis of sequences of *TraesTM9SF* genes.

### 2.3 TraesTM9SF motif structure analysis

The chromosomal positions of *TraesTM9SF* genes were obtained from the wheat genomic files. The online tool Multiple Expectation Maximization for Motif Elicitation MEME (https://meme-suite.org/meme/tools/meme, (Bailey et al., 2015)) was used for the prediction of conserved motifs of TraesTM9SF protein sequences. The number of motifs to be predicted for each sequence was set to 10 motifs. The images of conserved motifs of TraesTM9SF proteins were retrieved from the MEME results and merged using Adobe Photoshop software (https://www.adobe.com).

### 2.4 *Cis*-regulatory element analysis of *TraesTM9SF* genes

The upstream 1500 bp fragments of each of the *TraesTM9SF* genes were retrieved from the genomic DNA sequences since PlantCARE (Lescot et al., 2002) recommend to use the upstream 1500 bp sequence of the translation start site as a promoter of a given gene, and as it was performed previously by other researchers (Li et al., 2018; Huang et al., 2021). These sequences were submitted to the PlantCARE (Lescot et al., 2002) database (https://bioinformatics.psb.ugent.be/webtools/plantcare/html/) for predicting the cis-regulatory elements of the promotor sequences of *TraesTM9SF* genes. The visualization of cis-regulatory elements was achieved using the TBtools software (Chen et al., 2020). The counts of the cis-regulatory elements of *TraesTM9SF* genes were summarized and used for chord plot visualization with the chordDiagram function from the circlize package in R.

### 2.5 Synteny analysis for *TraesTM9SF* genes

Synteny analysis and gene duplication of *TraesTM9SF* genes were performed in TBtools (Chen et al., 2020). The MCScanX toolkit (Wang et al., 2012) was used for identifying syntenic gene pairs of putative *TM9SF* genes, and the Advance Circos option in the TBtools software (Chen et al., 2020) was used for visualization.

### 2.6 Protein-protein interaction network and functional enrichment analysis of TraesTM9SF proteins

The STRING tool (https://string-db.org/, (Szklarczyk et al., 2023)) was used for predicting the protein–protein interaction (PPI) network of TraesTM9SF proteins based on protein sequences with default parameters. The PPI network was downloaded and visualized in Cytoscape (https://cytoscape.org/, (Shannon et al., 2003)). The Gene Ontology (GO) and Kyoto Encyclopedia of Genes and Genomes (KEGG) annotation of TraesTM9SF protein sequences was achieved using the online eggNOG-mapper v2 (http://eggnog-mapper.embl.de, (Cantalapiedra et al., 2021)), and TBtools software (Chen et al., 2020) was used for their enrichment analysis.

### 2.7 Expression profiles of *TraesTM9SF* genes under nitrogen limitation conditions and reversal response induced by *B. amyloliquefaciens*

To investigate the potential role of *TraesTM9SF* genes in nitrogen limitation and their potential regulation by the growth-promoting bacterial species *B. amyloliquefaciens* PDR1 (GenBank accession number: OR858947.1), wheat was cultured under nitrogen limitation conditions and treated with the bacterial inoculum. Briefly, healthy plump wheat seeds (*T. aestivum* L. cv. Chinese Spring), bred and preserved in our laboratory, were surface sterilized with 70% ethanol and then soaked in aerated water for 24 hours. The seeds were sown in pre-sterilized plastic pots (20 cm diameter, 1.0 kg commercial vermiculite, 6 seeds per pot, repeated 8 times) containing autoclaved commercial vermiculite with 400 mL half-strength Hoagland’s nutrient solution (2 mM KNO3, 1 mM KH2PO4, 1 mM MgSO4, 1 mM CaCl2, 0.1 mM Fe-EDTA, 1 mM H3BO3, 1 mM ZnSO4, 0.5 mM CuSO4, 0.3 mM Na2MoO4, and 1 mM MnCl2) every 5 days. The medium was buffered at pH 6.0 using diluted 2 M NaOH. The wheat seedlings were randomly assigned to a constant temperature in controlled greenhouse growth chambers (SRG-260D, Hangzhou Shoulian Instrument CO.,LTD) at a temperature of 26 ± 2°C, humidity of 72 ± 2%, and light levels of 800 μmol m^−2^ s^−1^ from fluorescent tubes and tungsten lighting. Nitrogen limitation and strain treatments were started after the wheat reached the three-leaf stage. For nitrogen limitation treatment, KNO3 in half-strength Hoagland’s nutrient solution was replaced by KCl for 5 days of nitrogen starvation (NS) treatment. Strain treatment was by inoculating 1 mL of *B. amyloliquefaciens* PDR1 bacterial suspension culture at an optical density (OD) of 0.5 OD at 600 nm near the roots of each seedling or liquid LB medium which served as a control (Li et al., 2020). The treatment groups were as follows: Control (cultivation a control soil), Control+PDR1 (control soil + bacteria treatment), N limitation (cultivation on a low nitrogen soil), and N limitation + PDR1 (low nitrogen soil + bacteria treatment). For each treatment group, the following indexes were recorded from 12 different plants: flag leaf area, plant height, number of spikelets per spike, number of culms per plant, kernel weight per spike, flag leaf dry matter, number of kernels per spike, and spike dry matter. Moreover, the total nitrogen content in roots (12 roots per treatment group) was determined using a CHNS Analyzer (CHNS-932, LECO, USA).

### 2.8 RNA sequencing and cluster analysis for identification of significant genes

Finally, root samples from 12 different roots per treatment group were collected for whole transcriptome analysis of wheat grown in the above-mentioned conditions. Total RNA was extracted from the collected wheat root samples using the Invitrogen Plant RNA Purification kit (Carlsbad, CA, United States). The cDNA libraries were subsequently constructed, and after quality verification, the libraries were sequenced on the Illumina HiSeq 4000 apparatus as described in our previous studies (Gong et al., 2021). The RNA-seq reads underwent filtration to eliminate any low-quality reads and adaptor sequences, with a quality score threshold of Q > 20 being applied. The wheat genome was utilized as a reference for mapping the clean reads with the assistance of the TopHat spliced read aligner (Trapnell et al., 2012). The FPKM value was utilized via Cufflinks software to assess the expression levels of mRNAs (Trapnell et al., 2010). The expression matrix of *TraesTM9SF* genes was then extracted from the annotated sequencing data. Next, to identify the expression profiles of *TraesTM9SF* genes, Short Time-series Expression Miner (STEM, http://gene.ml.cmu.edu/stem/, (Ernst and Bar-Joseph, 2006)) was run on the transcriptome data. The pheatmap package was used for visualizing the expression profile of retrieved *TraesTM9SF* genes.

### 2.9 Quantitative real-time PCR

To validate the transcriptome data, we performed Quantitative Real-Time PCR (RT-qPCR). Total RNA isolation, reverse transcription, and PCR experiments were performed in triplicate as previously described (Gong et al., 2021), with the sequences of primers used summarized in **Table 1**. We employed the 2-ΔΔCt method (Livak and Schmittgen, 2001) to compute the relative gene expression of *TraesTM9SF* genes, using *GAPDH* (GenBank accession number: EF592180) as a reference housekeeping control.

**Table 1:** List of primers used in this study.

Gene Name	Sens	Primer sequences
***TraesTM9SF*-4**	F	5’-ACTTCATCCACAACCATTTGTC-3’
	R	5’-GGAGAGAGGAAACCCAGAACAG-3’
***TraesTM9SF*-5**	F	5’-ACCATTCAGCATCAAGCATC-3’
	R	5’-CCCACTTAATTTCACTCTCCTC-3’
***TraesTM9SF*-10**	F	5’-GCCACTTTTCTTGACATTCTCC-3’
	R	5’-GCTATTTTTCCCAGCTACACC-3’
***TraesTM9SF*-11**	F	5’-TCCCGCAGCAAAAATCTC-3’
	R	5’-ACAAGCAGCCAGTCAATATC-3’
***TraesTM9SF*-16**	F	5’-TCTCCATCCTCAACTCGCTC-3’
	R	5’-AACCCCATCACCAACCATC-3’
***TraesTM9SF*-17**	F	5’-TGGAAGTGGTGGTGGAAAG-3’
	R	5’-TGACGGACGAGAAGAGGTAG-3’
***TraesTM9SF*-20**	F	5’-AGCCAGCAATCAACCCCATC-3’
	R	5’-AACGGACGAGAAGAGGTAG-3’
***TraesTM9SF*-23**	F	5’-AAGAGGAGGCACAGGAGGAAAC-3’
	R	5’-AGCAATCAACCCCATCAGGAC-3’
***TraesTM9SF*-24**	F	5’-AAGAGGAGGCACAGGAGGAAAC-3’
	R	5’-AGCAATCAACCCCATCAGGAC-3’
***TraesTM9SF*-28**	F	5’-TGGAAGTGGTGGTGGAAAG-3’
	R	5’-TGGACGAAGTAGAACGAGAG-3’
***TraesTM9SF*-44**	F	5’-AAGAGGAGGCACAGGAGGAAA-3’
	R	5’-AATCAACCCCATCAGGACCCAG-3’
***GAPDH***	F	5’-CCACCAGCCGTCCCACAATA-3’
	R	:5’-GAACCAATCTCCCAATCCGTC-3’

### 2.10 Statistical analysis

For *in-silico* data, we generated boxplots using the ggplot2 package (Wickham et al., 2019) and used the T-test to gauge differences between paired groups. For experimental data, we detected differences among treatment groups by performing a one-way ANOVA analysis followed by Tukey’s *post hoc* multiple comparison test. We used the GraphPad Prism 9 software from GraphPad Software in San Diego, California, United States, for these analyses. A difference was considered significant at a p-value cutoff of 0.05.

## 3 Results

### 3.1 Identification and physiochemical properties of *TraesTM9SF* genes in wheat

**Figure 1:**
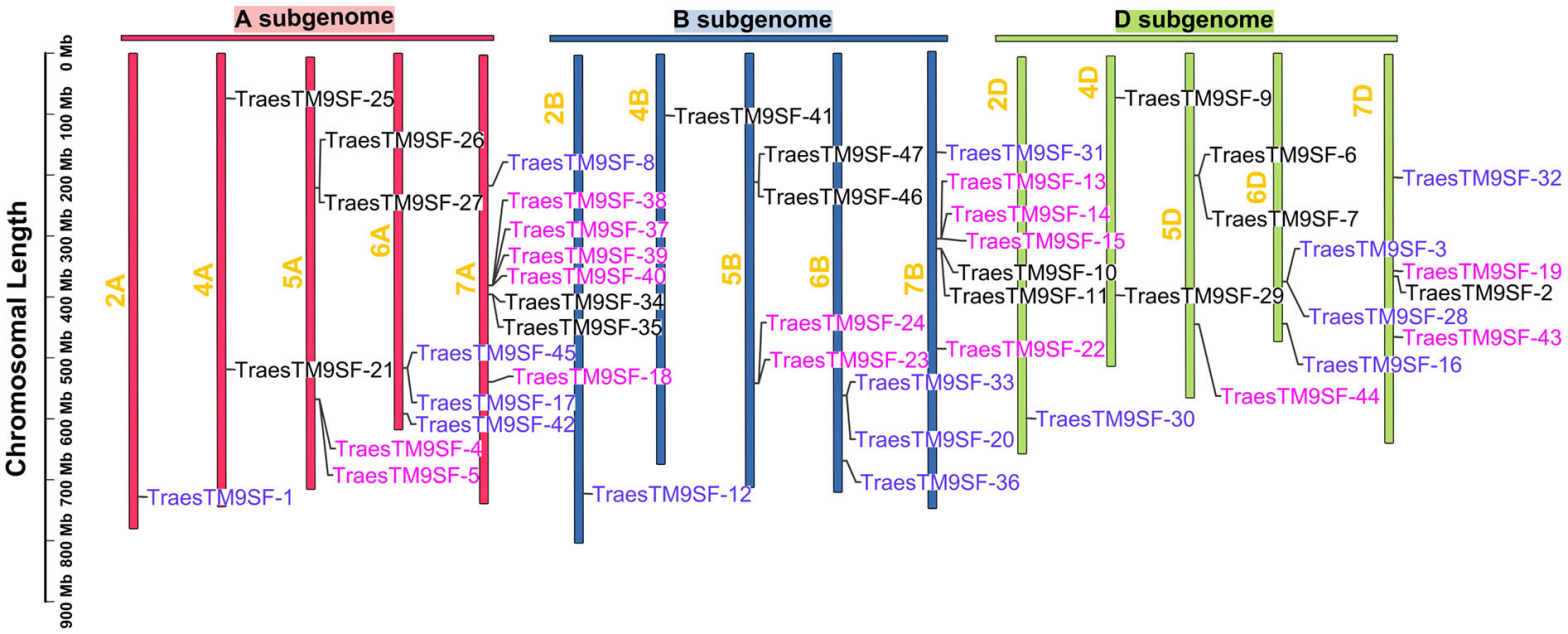
Chromosomal location of *TraesTM9SF* genes in the wheat genome.

A total of 47 *TraesTM9SF* genes in the wheat genome were retrieved by bioinformatical approaches. These putative wheat *TM9SF* genes were renamed from *TraesTM9SF-1* to *TraesTM9SF-47* based on their position in the phylogenetic tree. With the exception of chromosomes Un (which contain clone contigs that cannot be confidently placed on a specific chromosome), 1A, 1B, 1D, 3A, 3B, and 3D, the *TraesTM9SF* genes were unevenly distributed on the other wheat chromosomes (**Figure 1**). Specifically, they were found on chromosomes 2A, 2B, 2D, 4A, 4B, 4D, 5A, 5B, 5D, 6A, 6B, 6D, 7A, 7B, and 7D (**Figure 1**). The largest number of *TraesTM9SF* genes (8 genes) was recorded on chromosome 7A, accompanied by chromosome 7B (7 genes), chromosomes 7D, 5A, and 5B (4 genes on each), chromosomes 5D, 6A, and 6B (3 genes each), chromosomes 4A and 4D (2 genes each) and chromosomes 2A, 2B, 2D, and 4B (one gene each) (**Figure 1**). The detailed information on the 47 *TraesTM9SF* genes was summarized in **Supplementary Table S1**, and the protein sequences were given in **Supplementary Table S2**. Conserved Domain Search (CD search) confirmed that all of the *TraesTM9SF* proteins contained two domains, which were EMP70 and EMP70 superfamily domains (**Supplementary Table S3**). It is worth noting that EMP70 is a specific domain, while EMP70 superfamily domains encompass a broader group of related domains.

**Figure 2:**
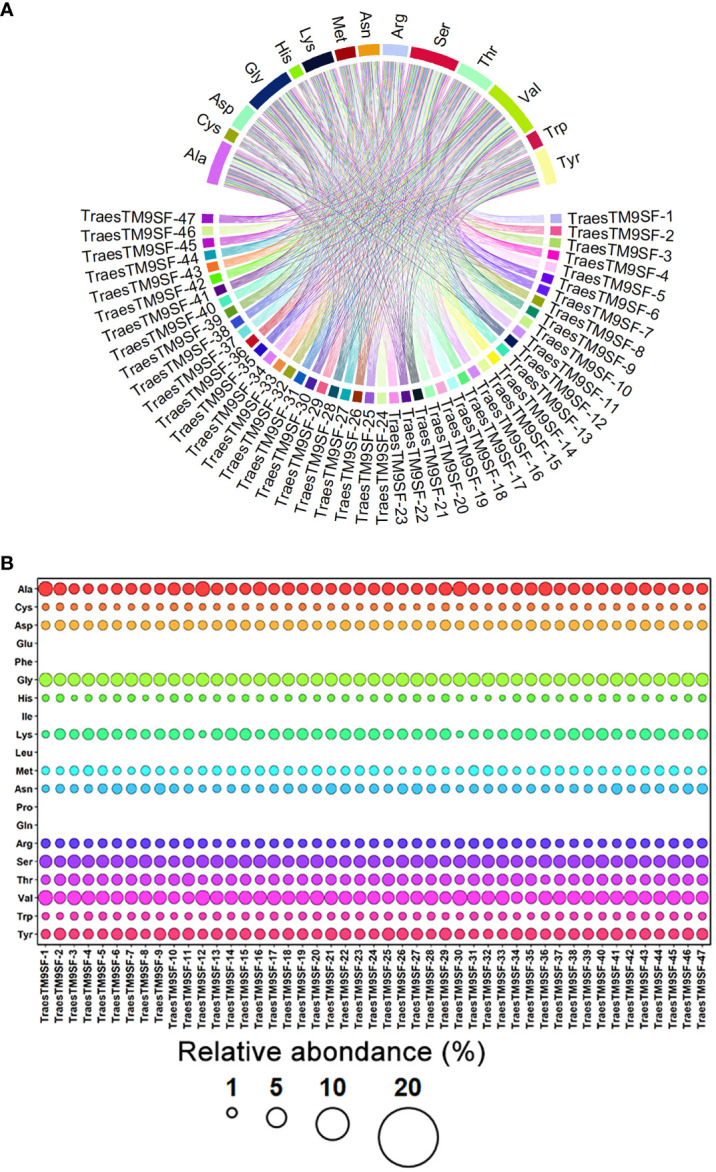
Amino acid composition of TraesTM9SF proteins. **(A)** Chord plot indicating the relationships between amino acids and different TraesTM9SF proteins. **(B)** Bubble plot indicating the relative abundance of amino acids in different TraesTM9SF protein sequences.

Physiochemical analysis indicated that the *TraesTM9SF* genes differed in their physicochemical properties, including protein length (294 - 467 amino acids) and molecular weight ranged from 32537.32 to 48189.04 Da (**Supplementary Table S1**). The low value of the instability index of TraesTM9SF proteins (instability index < 40) (**Supplementary Table S1**) indicated that these proteins are stable (Ikai, 1980). The aliphatic index of 38.57 to 65.61 (**Supplementary Table S1**) indicated that TraesTM9SF proteins have moderate to high thermostability while the Boman index ranged between 1.10 and 2.25 (**Additional File S1**) indicated a moderate cationic charge and a moderate binding ability of TraesTM9SF proteins to other proteins (De Cena et al., 2022). The theoretical charge from 18.14 to 33.87 (**Supplementary Table S1**) confirmed that TraesTM9SF proteins are positively charged or cationic while the isoelectric point of 9.42 - 10.24 (**Supplementary Table S1**) suggested that the TraesTM9SF proteins are relatively alkaline or basic in nature. The TraesTM9SF proteins with a GRAVY index range of -0.72 to 0.09 (**Supplementary Table S1**) indicated that these proteins are overall relatively hydrophilic. The abundance of amino acids composing TraesTM9SF proteins was as depicted in **Figure 2**.

### 3.2 Phylogenetic analysis of putative wheat *TM9SF* genes

**Figure 3:**
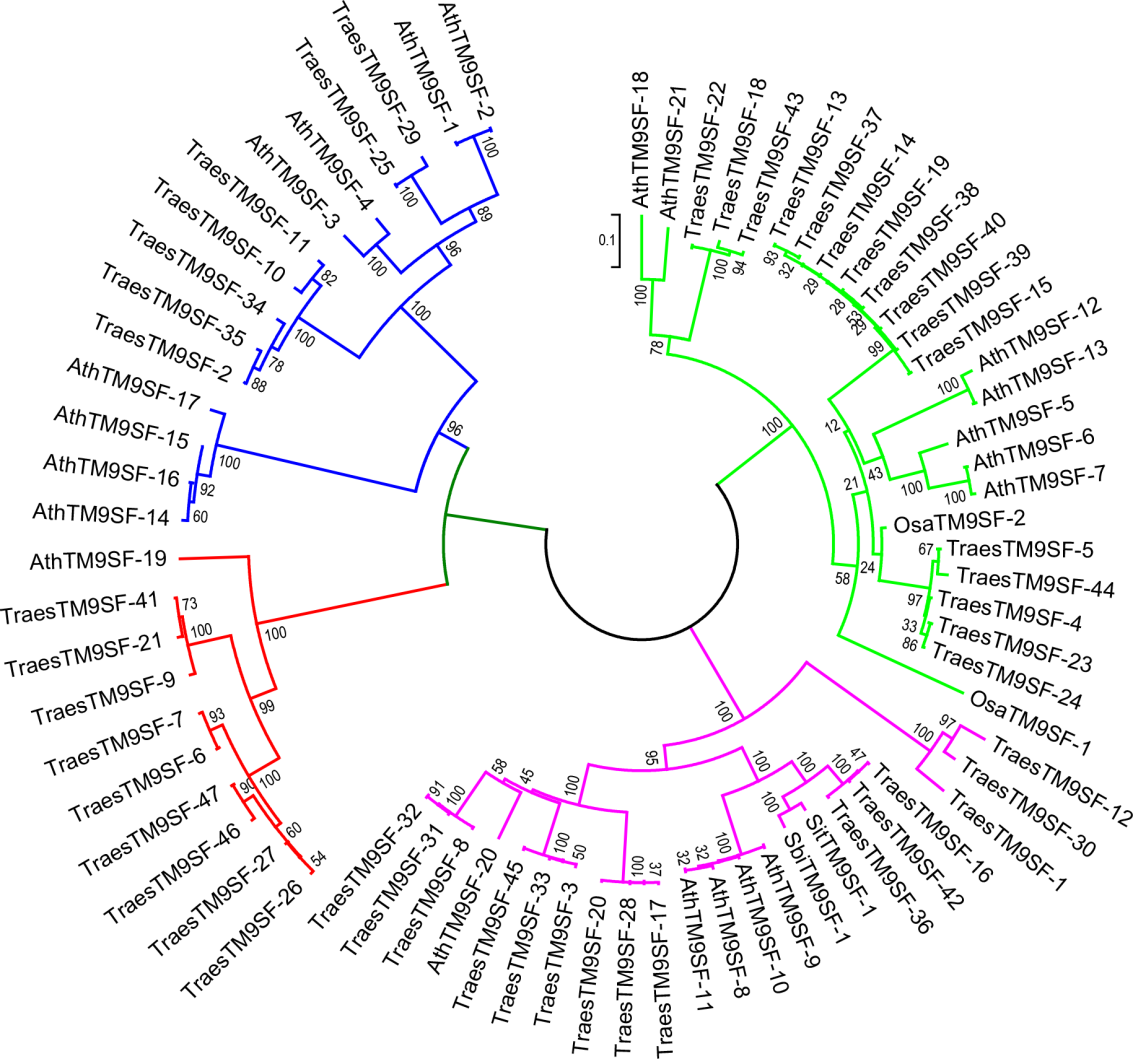
Neighbor joining phylogenetic tree of *OsTM9SF, SiTM9SF, SbTM9SF, AtTM9SF* and *TraesTM9SF* genes using MEGA.

The phylogenetic analysis of putative *SbiTM9SFs*, *SiTM9SFs*, *OsTM9SFs*, and *TraesTM9SF* genes indicated that these genes were clustered into four principal groups (**Figure 3**). There was an uneven distribution of *TraesTM9SF* genes in the different groups, and some of the *TraesTM9SF* genes were clustered with the *OsTM9SF, SiTM9SF, SbTM9SF*, and *AtTM9SF* genes (**Figure 3**). These results showed a potential phylogenetic relationship between the *TM9SF* genes. In addition, the clustering of *TraesTM9SF* genes with *TM9SF* genes of other species (*OsTM9SF, SiTM9SF*, and *SbTM9SF* genes) indicated a possible conservation of function among these genes.

### 3.3 Similarity and identity analysis of putative wheat *TM9SF* genes

To further understand the evolution of putative *TM9SF* genes in wheat, pairwise identity, and similarity analysis based on these genes was performed. The results (**Supplementary Table S5**) indicated that the similarity values ranged from 7.14% (*TraesTM9SF-20/TraesTM9SF-25*) to 100% (*TraesTM9SF-13/TraesTM9SF-37, TraesTM9SF-38/TraesTM9SF-19* and *TraesTM9SF-15/TraesTM9SF-39*). The lowest similarity of 4.76% was recorded between *TraesTM9SF-25* and *TraesTM9SF-20* genes, while the highest identity of 99.75% was obtained between the *TraesTM9SF-15* and *TraesTM9SF-39* genes (**Supplementary Table S5**).

### 3.4 Gene structure and motifs analyses of putative wheat *TM9SF* genes

**Figure 4:**
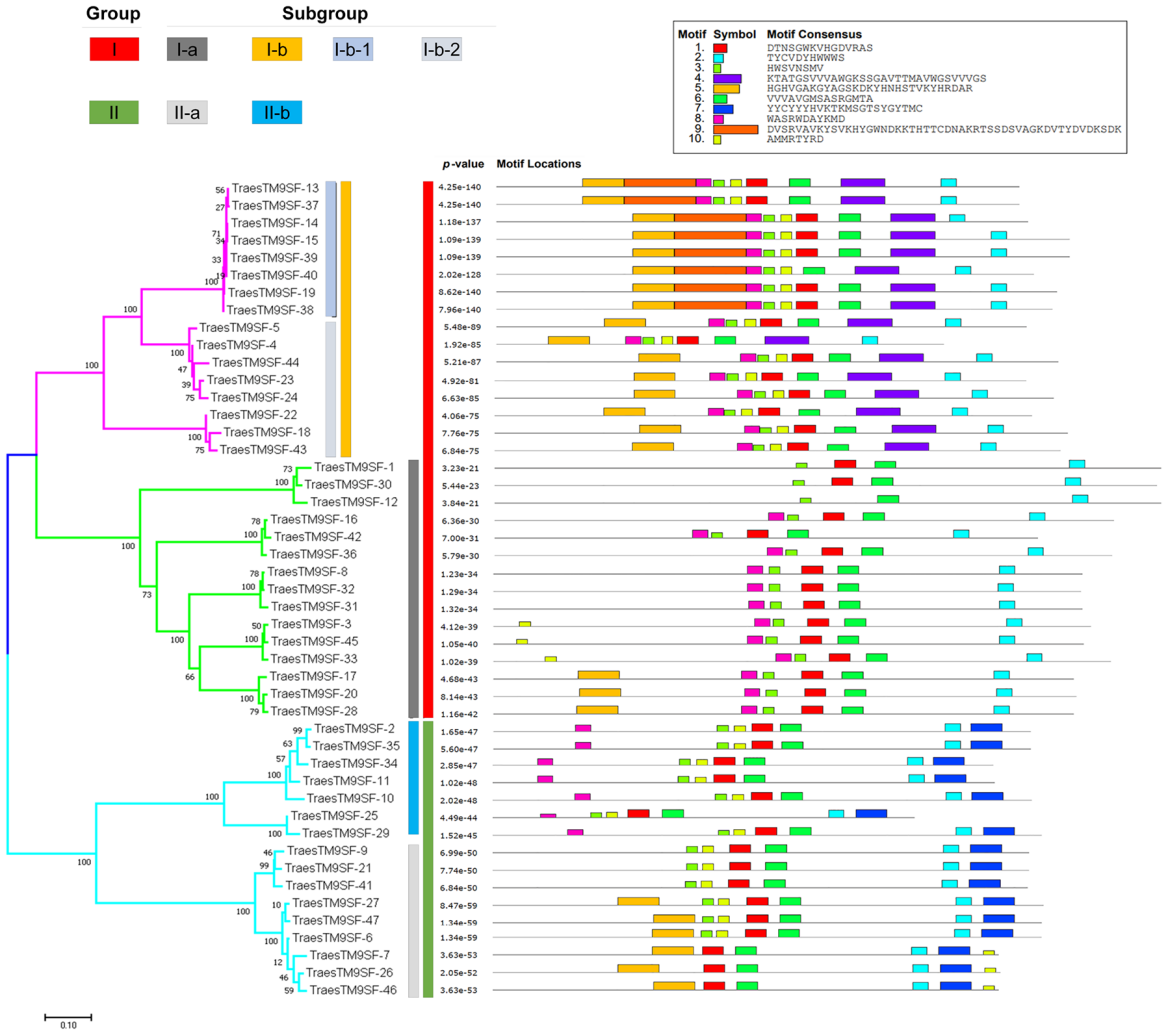
Motif structure and distribution on *TraesTM9SF* genes.

The relationship between the 47 *TraesTM9SF* genes was revealed via a phylogenetic tree, which was constructed using the NJ approach in MEGA7 (**Figure 4**). The fasta sequences of all *TraesTM9SF* proteins were summarized in **Supplementary Table S4**. Based on the phylogenetic relationship, *TraesTM9SF* proteins were divided into two main clusters in the phylogenetic tree (**Figure 4**). Group I was the largest group and contained 31 *TraesTM9SF* genes, while Group II contained 16 genes (**Figure 4**). Group I was divided into two subgroups, I-a and I-b; subgroup I-b was further divided into I-b-1 and I-b-2 (**Figure 4**). Group II was divided into subgroup II-a and subgroup II-b. In addition, the analysis of conserved motifs among the 47 *TraesTM9SF* genes by the Multiple Expectation Maximization for Motif Elicitation (MEME) (Bailey et al., 2009) tool indicated that each group or subgroup had a specific pattern of motifs (**Figure 4**). The *TraesTM9SF* genes in Group I were characterized by the absence of motif 7, which was present in the sequences of *TraesTM9SF* genes in Group II (**Figure 4**). The sequences in subgroup II-a were characterized by the presence of motif 5 and the absence of motif 8, while the proteins in the subgroup II-b were characterized by the absence of motif 5 and the presence of motif 8 (**Figure 4**). In Group I, the subgroup I-a was characterized by the absence of motif 4, while the subgroup I-b was characterized by the presence of this motif (**Figure 4**). In addition, the subgroup I-b-1 could be characterized by the presence of motif 9, while the subgroup I-b-2 was devoid of this motif (**Figure 4**). The motifs 1, 2, 3, and 6 were present on almost all of the *TraesTM9SF* genes (**Figure 4**), indicating a conserved gene structure. These observations demonstrated that *TraesTM9SF* genes in a given group share highly similar gene structures and the presence or absence of specific motifs can be used to classify *TraesTM9SF* genes into distinct subgroups. This was corroborated by their phylogenetic relationships (**Figure 4**).

### 3.5 *Cis*-regulatory element analysis of *TraesTM9SF* genes

**Figure 5:**
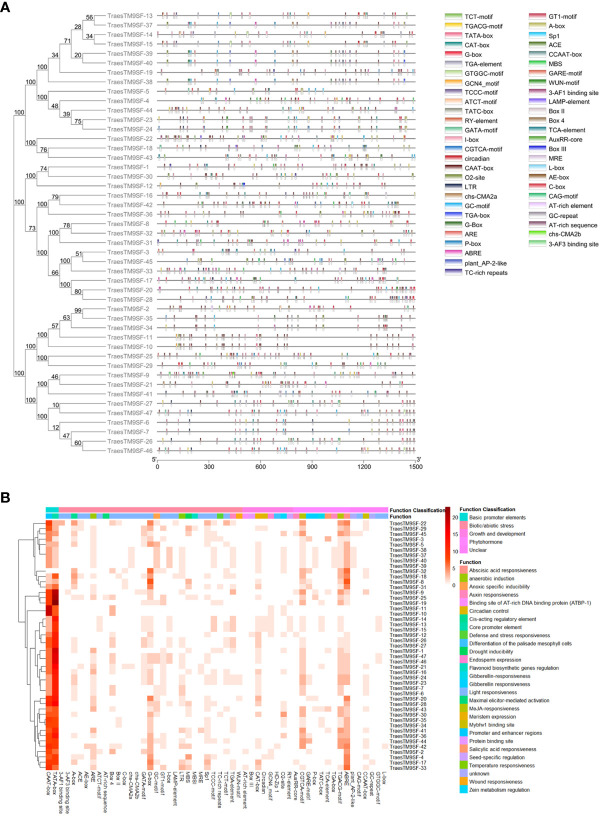
*Cis*-regulatory elements of *TraesTM9SF* genes. **(A)** Distribution of *Cis*-regulatory elements on *TraesTM9SF* Genes. **(B)** Heatmap showing the abundance of *Cis*-regulatory elements of *TraesTM9SF* genes.

The 1500 bp upstream promoter region of each of the translated region of putative *TM9SF* genes in wheat was used to analyze the *cis*-regulatory element analysis in order to understand the role of *TraesTM9SF* genes. The results indicated that 54 *cis*-regulatory elements were present in the *TraesTM9SF* promoter regions (**Figure 5**). The light responsiveness category was the most represented and consisted of 22 *cis*-acting elements, including 3-AF1, 3-AF3, GT1-motif, Sp1, MBS, MBSI, MRE, CCAAT-box, ATCT-motif, Box 4, CAG-motif, chs-CMA2b, GATA-motif, GTGGC-motif, I-box, L-box, LAMP-element, TCCC-motif, TCT-motif, Box II, chs-CMA2a, and the AE-box *cis*-elements (**Figures 5A, B**). The phytohormone responsive category contained 9 *cis*-acting elements including TGA-element, ABRE, AuxRR-core, CGTCA-motif, TGACG-motif, P-box, GARE-motif, and TGA-box (**Figures 5A, B**) while the biotic/abiotic stress-related *cis*-elements were 29 and included WUN-motif cis-element, MBS, GC-motif, ARE, LTR, and TC-rich repeats (**Figures 5A, B**). The plant growth and development category comprised of 8 *cis*-acting elements, namely AT-rich element, Box III, CAT-box, Circadian, GCN4_motif, HD-Zip 1, O2-site, and RY-element (**Figures 5A, B**).

### 3.6 Synteny analysis of *TraesTM9SF* genes

**Figure 6:**
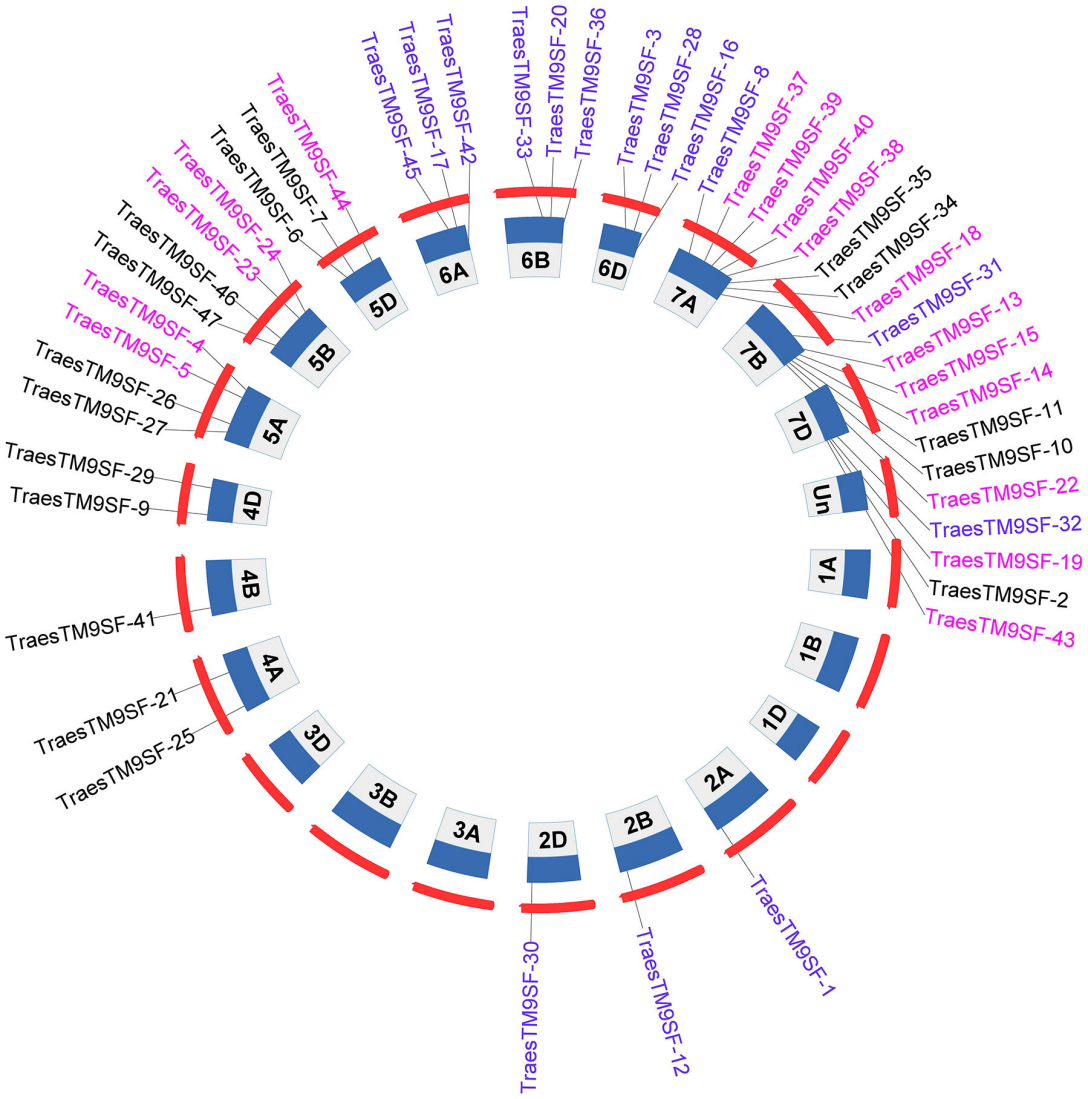
Synteny analysis of *TraesTM9SF* genes. Circos plot was used to plot gene duplications. The plot indicated no duplication of *TraesTM9SF* Genes.

Synteny analysis was performed to further understand the evolution and expansion mechanism of the *TraesTM9SF* gene family in the wheat genome. *TraesTM9SF* gene duplications were assessed based on a tandem or segmental duplication. The gene duplication analysis results indicated no *TraesTM9SF* gene pair (**Figure 6**), showing no duplication of *TraesTM9SF* genes in the wheat genome.

### 3.7 Prediction of transcription factor regulatory network of *TraesTM9SF* genes and functional analysis

**Figure 7:**
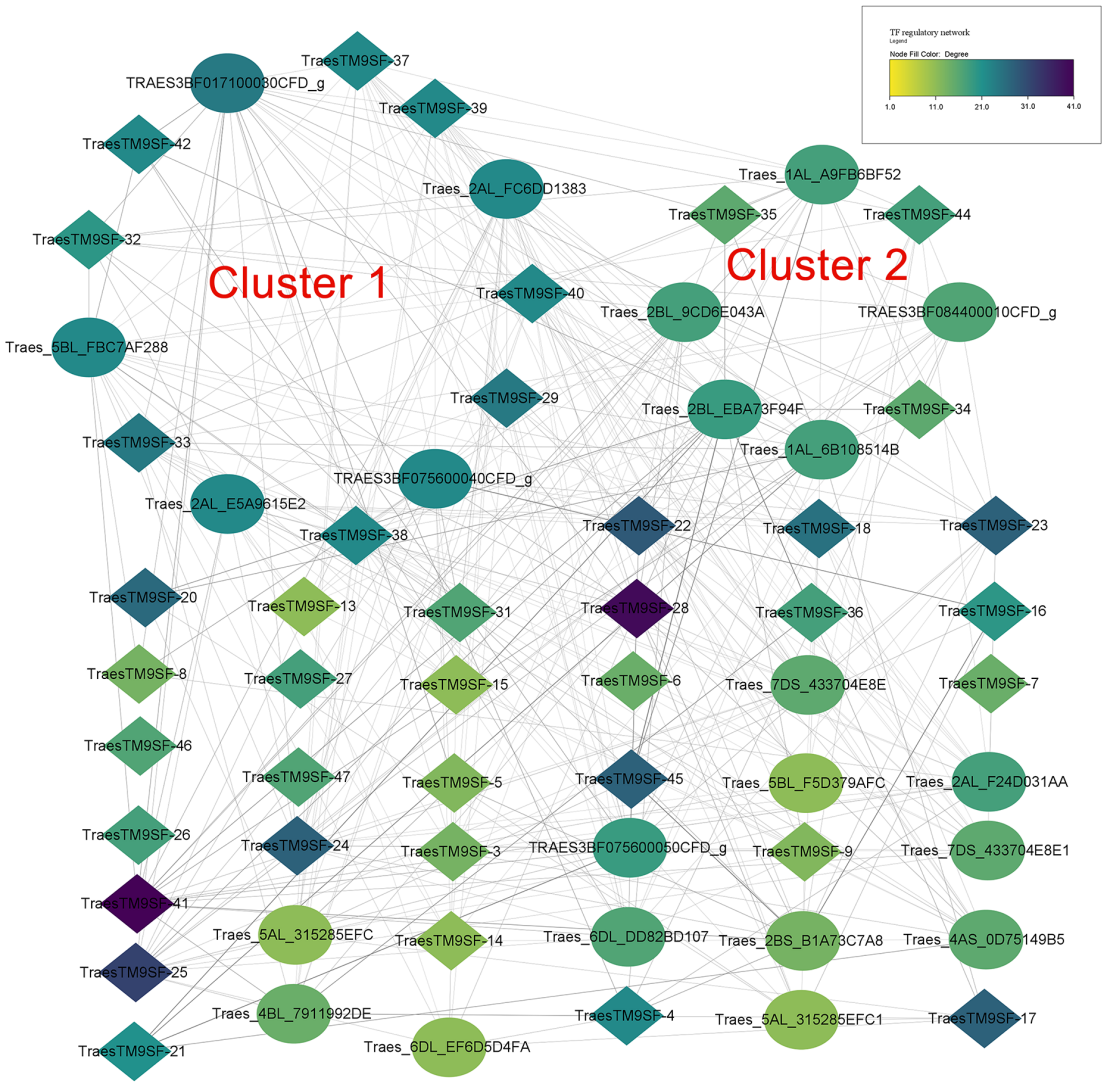
Regulatory network of TraesTM9SFs and their TFs. Diamonds indicate genes while the ellipses indicate TFs.

**Figure 8:**
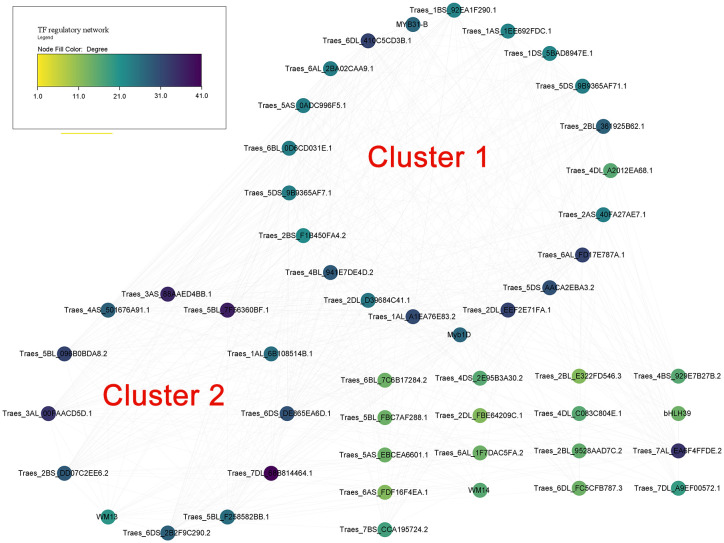
Protein-protein interaction (PPI) network of TraesTM9SFs and their TFs.

The prediction of potential TFs targeting the 1500bp upstream region of the translated regions of putative wheat *TM9SF* genes indicated 776 regulations between 177 TFs and 47 genes. In addition, 98 TFs targeted *TraesTM9SF* genes in the input gene set under the cutoff of p-value <0.05. **Figure 7** depicts the regulatory interactions between *TraesTM9SF genes* (in diamonds) and their TFs (in ellipses). Additionally, **Figure 8** depicts the protein-protein interaction (PPI) network of *TraesTM9SF genes* and their TFs. The PPI network analysis indicated that the network comprised 138 nodes and 772 edges with an average number of neighbors of 11.188 (**Figure 8**). The functional enrichment of the TFs and TraesTM9SF proteins in the PPI network indicated that these proteins were functionally enriched in the biological processes of regulation of transcription, regulation of gene expression, regulation of molecular function, and other development-, metabolism- and cell-related processes (**Supplementary Data Sheet S1**). The enriched cellular components were cellular anatomical entity, nucleus, intracellular, and host cell nucleus. At the same time, the most represented molecular functions were DNA binding, DNA-binding transcription factor activity, sequence-specific DNA binding, and heterocyclic compound binding (**Supplementary Data Sheet S1**). The enriched pathways were plant hormone signal transduction and circadian rhythm of plants (**Supplementary Data Sheet S1**). The result of GO terms associated with TraesTM9SF proteins obtained from the eggNOG-mapper was depicted in **Supplementary Data Sheet S2**.

### 3.8 Expression profiles of *TraesTM9SF* genes under nitrogen limitation condition and effect of PDR1

**Figure 9:**
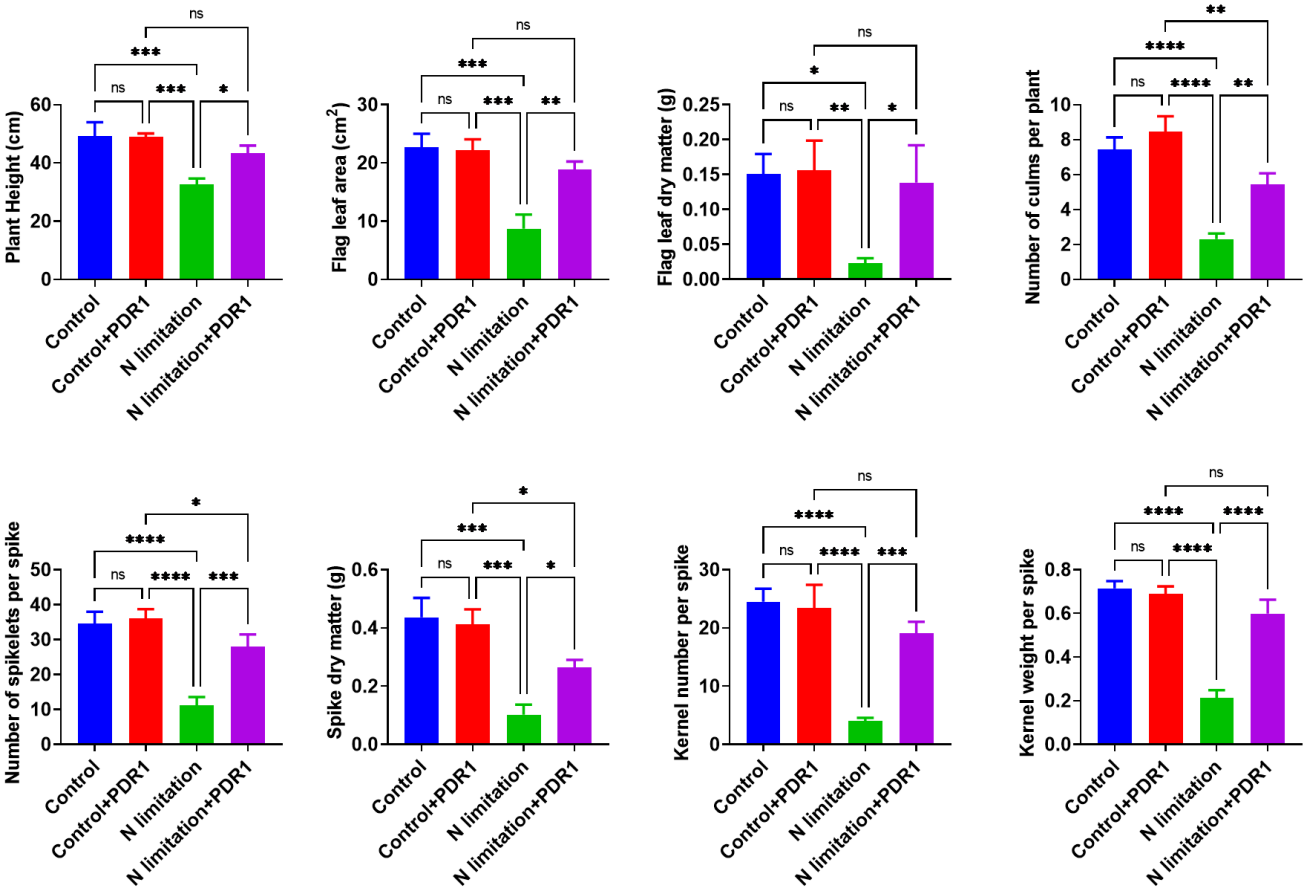
Determination of different indexes under stress and different treatment conditions. *p<0.05, **p<0.01, ***p<0.01 and ****p<0.01 among the compared groups; ns stands for non-significant. A total of n=12 plants per group were used and the experiments were performed in triplicate. The results are expressed as mean ± SD.

**Figure 10:**
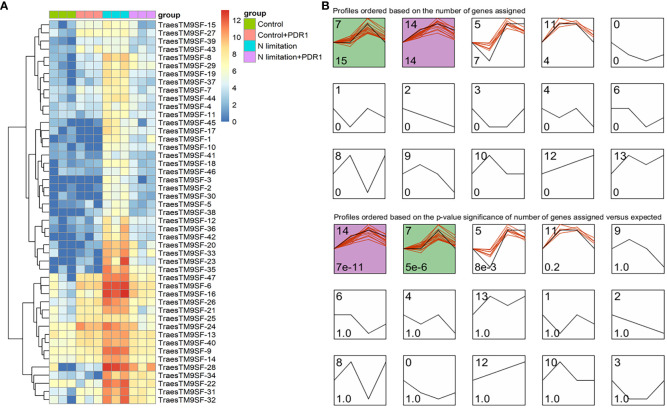
Transcriptome expression profile of *TraesTM9SF* genes by RNA sequencing under nitrogen limitation and *B amyloliquefaciens* PDR1 conditions. **(A)** Heatmap showing the expression profile of *TraesTM9SF* genes under different treatment conditions. **(B)** Clustering of *TraesTM9SF* genes based on their expression levels under different treatment conditions. The number in the upper right of each box corresponds to the profile number representing this cluster or group of genes while the number in the lower right indicate either the number of genes (upper panel) within each profile or the p-values (lower panel) associated with each profile.

**Figure 11:**
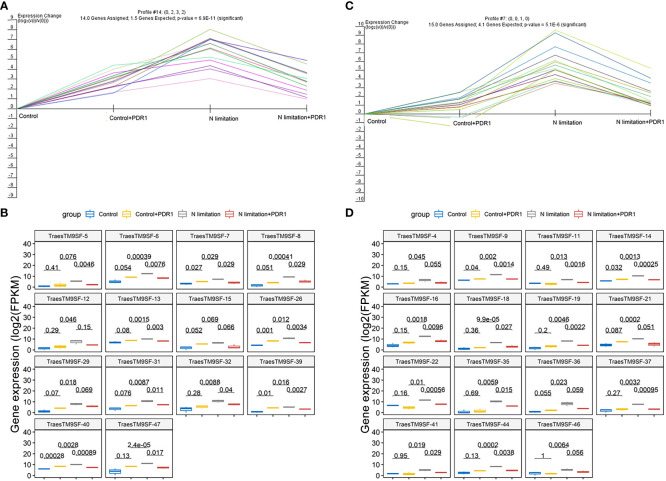
Transcriptome expression profile of *TraesTM9SF* genes in cluster 7 and cluster 14. **(A)** The expression profile of *TraesTM9SF* genes on cluster 7 under different treatment conditions. **(B)** Boxplots showing the expression profile changes of *TraesTM9SF* genes on cluster 7 under different treatment conditions. The numbers in each box represent the p-values obtained by T-test analysis among the indicated groups. **(C)** The expression profile of *TraesTM9SF* genes on cluster 14 under different treatment conditions. **(D)** Boxplots showing the expression profile changes of *TraesTM9SF* genes on cluster 14 under different treatment conditions. The numbers in each box represent the p-values obtained by T-test analysis among the indicated groups.

The functional characterization of *TraesTM9SF* genes has not been studied in wheat. To investigate the potential involvement of the functional role of *TraesTM9SF* genes in nitrogen starvation conditions and the effect of *B. amyloliquefaciens* PDR1, the wheat seedlings were cultivated in nitrogen limitation or combined treatment of nitrogen limitation and PDR1 strain and their relative controls. Nitrogen starvation led to deleterious changes in the plant traits (**Figure 9**). The growth of the wheat plant was severely affected in nitrogen limitation conditions; the measured indexes varied considerably among plants grown in normal and nitrogen limitation conditions (**Figure 9**). Specifically, the plants grown under nitrogen limitation conditions were shorter, and their flag leaf area and flag leaf dry matter were decreased compared to those in the normal conditions (**Figure 9**). In general, plants in the nitrogen limitation group had a decreased number of culms, decreased number of spikelets per spike, lower spike dry matter, lower kernel number per spike, and lower kernel weight per spike compared to the control plants (**Figure 9**). Moreover, the treatment with the bacterial strain PDR1 did not affect the plants grown under normal conditions but counteracted the deleterious effect of nitrogen starvation (**Figure 9**). Subsequently, to explore the effect of nitrogen starvation on the root transcriptome of the plant, the root samples were subjected to RNA sequencing. The heatmap showing the expression of *TraesTM9SF* genes in the root system of the wheat was as depicted in **Figure 10A**. The time series clustering was subsequently performed, and the results showed that 2 significant clustering profiles could be obtained (**Figure 10B**). The first cluster (profile 14) comprised 14 genes whose expression levels were increased by both the PDR1 strain and N limitation treatments with a pronounced effect for the N limitation treatment. The combined treatment with the PDR1 strain and N limitation also reversed the effect of N limitation treatment on the expression of these genes (**Figures 10B**, **11A, B**). Interestingly, the second significant cluster (profile 7) was characterized by the upregulation of *TraesTM9SF* genes under nitrogen limitation conditions, but this trend was reversed by the treatment with the growth-promoting bacterial strain *B. amyloliquefaciens* PDR1 (**Figures 10B**, **11C, D**; **Supplementary Data Sheet S3**). The genes in both profiles were considered as candidate nitrogen limitation- and PDR1-responsive *TraesTM9SF* genes (**Supplementary Data Sheet S4**). The boxplots in **Figures 11B, D** indicated significant differences in the expression of these genes in pairwise comparisons of treatment groups. Overall, these results showed that *TraesTM9SF* genes are expressed in the root system of wheat and are possibly involved in the response of this plant to biotic and abiotic stress conditions.

### 3.9 Validation of RNA-seq expression data by quantitative real-time polymerase chain reaction

**Figure 12:**
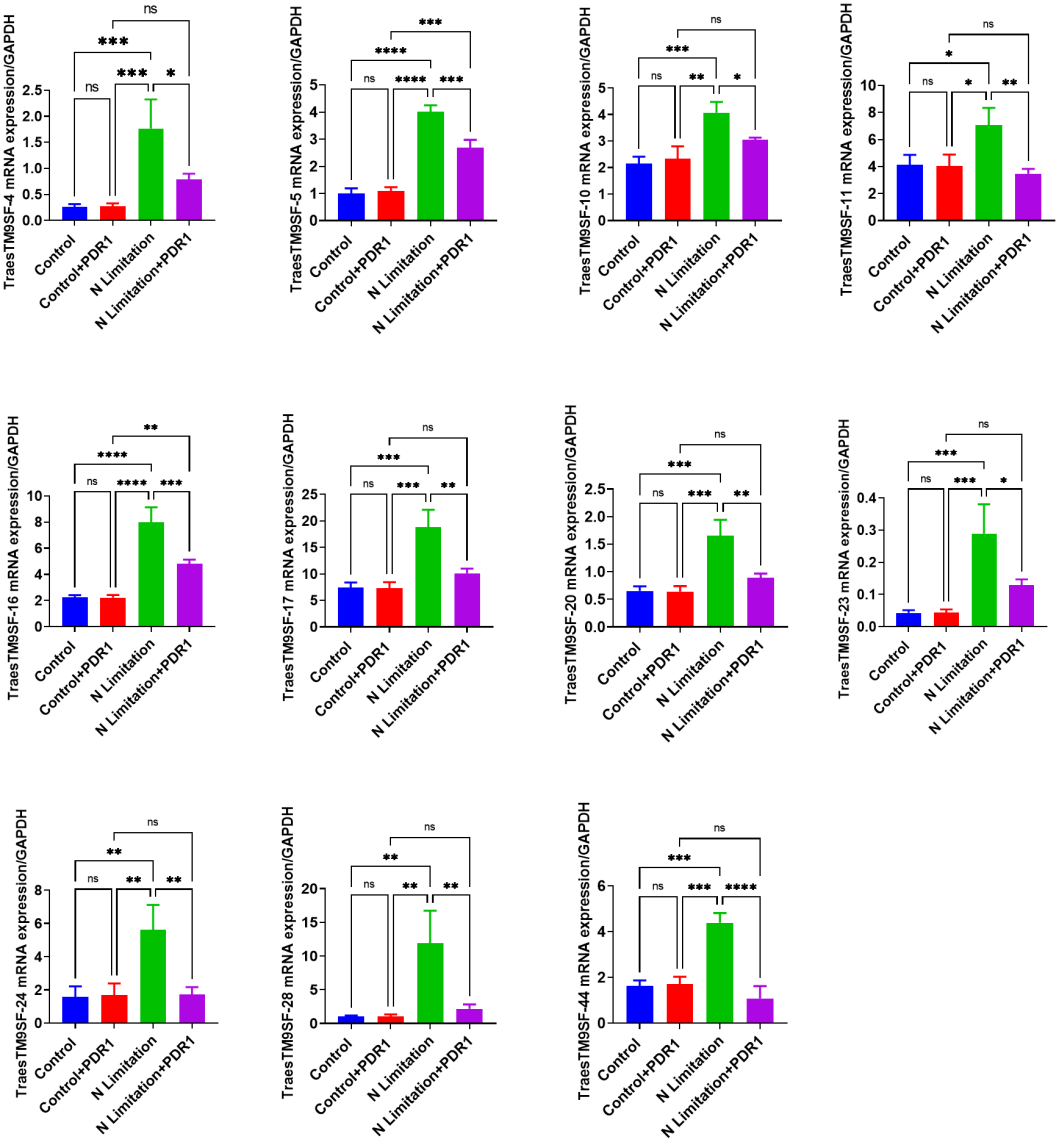
qRT-PCR validation of the expression of PDR1-responsive *TraesTM9SF* genes from transcriptome data. *p<0.05, **p<0.01, ***p<0.01 and ****p<0.01 among the compared groups; ns stands for non-significant. A total of n=12 plants per group were used and the experiments were performed in triplicate. The results are expressed as mean ± SD.

The TraesTM9SF*-4, TraesTM9SF-5, TraesTM9SF-10, TraesTM9SF-11, TraesTM9SF-16, TraesTM9SF-17, TraesTM9SF-20, TraesTM9SF-23, TraesTM9SF-24, TraesTM9SF-28*, and *TraesTM9SF-44* genes were chosen for validation by qRT-PCR. After normalization with the *GAPDH* reference gene, the analyzed *TraesTM9SF* genes showed expression trends similar to that of the RNA-Seq data (**Figure 12**). These results showed that RNA-Seq was credible and confirmed the functional role of *TraesTM9SF* genes in nitrogen limitation conditions and their potential involvement in the beneficial effect of PDR1.

## 4 Discussion

This study identified 47 *TraesTM9SF* genes with uneven chromosomal distribution in the wheat genome. The putative *wheat* TM9SF proteins contained the EMP70 and EMP70 superfamily domains and exhibited dissimilar physicochemical properties, though similarities and identities could be recorded among TraesTM9SF proteins. In addition, 37 of these *TraesTM9SF* proteins were most likely located in the extracellular region, whereas the other ten were located in the nucleus. The *TraesTM9SF* genes shared a phylogenetic relationship with the *OsTM9SF, SiTM9SF, SbTM9SF*, and *AtTM9SF* genes. Moreover, phylogenetic differences were found between *TraesTM9SF* genes based on the conserved motifs found on these genes. Fifty-four *cis*-regulatory elements were identified in the *TraesTM9SF* promoter regions and were mainly associated with light responsiveness, hormone response, and drought-inducibility. The gene duplication analysis results indicated no *TraesTM9SF* gene pair, showing no duplication of *TraesTM9SF* genes in the wheat genome. Moreover, 177 TFs regulating 47 *TraesTM9SF* genes were identified and formed a complex regulatory network with TM9SF proteins. Transcriptome analysis and validation by qRT-PCR indicated that the *TraesTM9SF genes* are expressed in the root system of wheat and may be potentially involved in the response of this plant to *B. amyloliquefaciens* PDR1 and nitrogen limitation conditions.

In this study, the total number of *TraesTM9SF* genes in wheat was 47, which was higher than that of *A. thaliana* (21 genes), rice (2 genes), *S. bicolor* (one gene), and *S. italica* (one gene). The higher number of *TM9SF* genes in wheat, compared to the other crops, might be due to functional importance, genetic variations, environmental adaptation or regulatory mechanisms. This finding also indicated the expansion, abundance, and potential functional diversity of the *TraesTM9SF* gene family in wheat. This result was in line with the findings that despite the vast number of familial genes in plant genomes, lineage-specific expansions might lead to significant discrepancies in the size of a given gene family among plant species (Panchy et al., 2016). For instance, the protein kinase superfamily has 2,532 members in *Eucalyptus grandis* and 426 in *Chlamydomonas reinhardtii* (Panchy et al., 2016). Moreover, the considerable number of *TraesTM9SF* genes in wheat relative to other plant species can be imputable to the complex genetic material of the wheat (Brenchley et al., 2012; Chapman et al., 2015). Polyploidy events, such as genome duplication, have been demonstrated to contribute substantially to gene family expansion and functional heterogeneity in plants (Soltis et al., 2015; Fang et al., 2022b; Mao et al., 2022). The large-scale repertoire of *TraesTM9SF* genes in wheat can supply the crop with various functional capacities, enabling it to adapt to various ecological niches and endure diverse abiotic and biotic stresses (Pont et al., 2013). Previous studies highlighted the pivotal role of *TM9SF* genes in abiotic stress responses, including drought, salinity, and heat stress (Hossain et al., 2016; Banik and Dutta, 2023). Therefore, the ability to cope with environmental challenges may be improved by the high number of *TraesTM9SF* genes in wheat.

Gene duplication plays a significant role in genome evolution (Magadum et al., 2013). Synteny and collinearity analyses indicated no gene duplication of *TraesTM9SF* genes in wheat genes, indicating that the *TraesTM9SF* genes might not be involved in the evolution of the wheat genome or that its implication in the genome evolution may be driven via other mechanisms. It can also be concluded that the evolutionary mechanism of the *TraesTM9SF* gene showed maintenance during the domestication of wheat. The finding, however, is contrary to the results of earlier studies, which have reported that gene duplications are a vital factor for genetic family expansion and plant function diversification (Fang et al., 2022b; Mao et al., 2022). The lack of gene duplication events in wheat *TraesTM9SF* genes poses exciting questions. Segmental or whole-genome duplications, in which wheat has undergone genetic modification over time, may have played a role in the expansion of the *TraesTM9SF* gene family in wheat (Panchy et al., 2016). Moreover, the *TraesTM9SF* gene family in wheat could have multiple copies due to retention or acquisition from related species via HGT (Dean et al., 2018; Murphy et al., 2019).

Conserved motifs play pivotal roles in the function of genes harboring them and are implicated in DNA binding, transcriptional activity, and protein interaction (Orozco and Hanley-Bowdoin, 1998). They are also involved in diverse plant functions in response to biotic and abiotic stresses (Singh and Graether, 2020). Herein, we discovered that the *TraesTM9SF* genes had gene structures characterized by the presence of conserved motifs, and some of these motifs were present in all of the *TraesTM9SF* genes. The conserved motifs are a critical functional element that can provide insight into potential roles and functional characteristics of genes (Bailey et al., 2009). Our study found conserved motifs in the *TraesTM9SF* gene family, indicating that certain functional domains or regions are preserved across genes. This conservation implies that these motifs can play a crucial role in the structure, function, and regulation of *TraesTM9SF* genes. Similar findings have been observed in other gene families where conserved features were associated with specific functional domains and protein interactions (Robson et al., 2001; Shoemaker et al., 2006).

*Trans*- and *cis*-regulatory elements are the two regulatory systems plants use to regulate gene expression (Vandepoele et al., 2009; Biłas et al., 2016; Brkljacic and Grotewold, 2017; Huang and Ecker, 2018; Ricci et al., 2019; Wang et al., 2019). The *cis*-regulatory elements in the promoter region are implicated in the tissue-specific, stress-responsive, and various stimulus-responsive genes (Sheshadri et al., 2016; Luján et al., 2021). In this study, we predicted 54 *cis*-acting elements in the promoter region of *TraesTM9SF* genes. These *cis*-regulatory components, which are mainly found to be related to the sensitivity of the light, suggested that *TraesTM9SF* genes could play an essential role in lighting-mediated processes like photosynthesis, photomorphogenesis, and melatonin regulation (Tan et al., 2014; Hwang et al., 2020; Zeng et al., 2022). This finding is consistent with previous studies suggesting the role of *cis*-acting elements in regulating light-responsive genes (Zhai et al., 2019). The *cis*-elements related to development, phytohormones, and metabolism were also identified, indicating the potential role of *TraesTM9SF* genes in hormonal regulation, development, and metabolic processes in wheat. This suggests that the *TraesTM9SF* genes play an important role in various developmental processes, hormone signaling pathways, and metabolic regulation in wheat. This observation aligns with the roles of various gene families involved in plant evolution, development, and metabolism (Jiang et al., 2015). *Cis*-acting elements involved in biotic and abiotic stresses, such as defense, stress, drought, and salinity, were also predicted, suggesting the potential of *TraesTM9SF* genes in regulating the response of wheat to biotic and abiotic stress, which requires further experimental validation in future studies. Thus, the *TraesTM9SF* genes may regulate plant response to environmental challenges and stress tolerance. The presence of stress-responsive *cis*-acting elements in the promoter regions of genes has been reported in several stress-responsive gene families, highlighting their importance in regulating stress-responsive gene expression (Zou et al., 2011; Sheshadri et al., 2016).

Since the role of *TraesTM9SF* genes has not been confirmed in wheat, we cultivated the wheat under *B. amyloliquefaciens* PDR1 and nitrogen limitation conditions and performed transcriptome sequencing to explore their expression patterns. We found that the *TraesTM9SF* genes were expressed in the root system of the wheat under normal cultivation conditions. Our results showed that in the root system, *TraesTM9SF* genes are expressed and seem likely to play an important role in plant growth, uptake of nutrients, or other root-related processes. After treatment with nitrogen limitation conditions, upregulation in the expression of *TraesTM9SF* genes was observed, indicating that these genes are responsive to nitrogen limitation. Thus, *TraesTM9SF* genes may play an important role in the mechanisms underlying the effect of nitrogen limitation on wheat, for example, nitrogen uptake, conversion, or recycling processes, which are needed for nutrient homeostasis and maintenance of plant growth under reduced nitrogen availability (Masclaux-Daubresse et al., 2010; Muratore et al., 2021; Zhang et al., 2023). Interestingly, the potential role of the alterations of *TraesTM9SF* genes due to a lack of nitrogen in increasing wheat resistance to nutrient stress, as well as optimizing nitrogen use for increased crop growth and productivity is apparent from the observations of this study. Moreover, the co-cultivation of wheat *B. amyloliquefaciens* PDR1 strain under nitrogen limitation conditions led to downregulation of *TraesTM9SF* genes, suggesting possible regulation of these genes by the beneficial bacterium under nutrient-stress conditions.

Moreover, though the PDR1 strain has been reported to be beneficial for *A. thaliana* (Li et al., 2020), our study is the first to report its role in reversing nitrogen limitation-induced gene dysregulation in wheat. Therefore, our work about *TraesTM9SF* genes in wheat has potential applications for crop breeding. The expression patterns of these genes in the root system, their response to nitrogen limitation, and their interaction with the *B. amyloliquefaciens* PDR1 strain suggest their potential roles in root development, nutrient stress responses, and plant-microbe interactions. Further functional characterization and molecular studies are required in wheat under various stress conditions to determine the precise mechanisms and biological function of *TraesTM9SF* genes.

Our study has some limitations. Although the transcript levels of some *TraesTM9SF* genes were responsive to nitrogen limitation and despite *B. amyloliquefaciens* PDR1 treatment conditions indicated that these genes might play important roles in response to nitrogen limitation or *B. amyloliquefaciens*, further experimental evidence is still needed to confirm their functional roles in wheat under these processes. In our future studies, we will screen one or several potential genes of the *TraesTM9SF* family in the RNA-seq data of wheat roots treated by nitrogen deficiency and bacterial infection and perform functional characterization of these potential genes to validate their roles in nitrogen utilization and resistance/exposure to *B. amyloliquefaciens* PDR1.

## 5 Conclusion

TM9SF proteins play important roles in the growth and development of plants; however, there has yet to be a relevant report on *T. aestivum*. In our study, the identification and expression profiling of putative *TM9SF* genes in *T. aestivum* was performed. Several bioinformatics analyses were carried out that include the identification of DNA/protein sequences, chromosomal localization, gene structure, calculation of genomic duplications, determination of phylogenetic groups, examination of protected motif regions, and identification of gene ontology categories. The expression patterns of *TM9SF* genes under nitrogen limitation and *A. amyloliquefaciens* PDR1 conditions were also verified experimentally. These results are essential for providing good candidate genes, which will be used for potentially breeding wheat varieties with high resistance to nitrogen limitation and response to nutrients via genetic engineering. Thus, our study provides a comprehensive basis for molecular and evolutionary characterization of *TraesTM9SF* genes in wheat and their role in wheat response to nitrogen limitation or *B. amyloliquefaciens*.

## Data availability statement

The data that support the findings of this study have been deposited into the CNGB Sequence Archive (CNSA) of China National GeneBank DataBase (CNGBdb) with accession number CNP0003662 (https://db.cngb.org/search/project/CNP0003662/).

## Author contributions

FL: Conceptualization, Data curation, Formal Analysis, Funding acquisition, Investigation, Methodology, Project administration, Resources, Software, Supervision, Validation, Visualization, Writing – original draft, Writing – review & editing. KX: Conceptualization, Data curation, Formal Analysis, Investigation, Methodology, Software, Validation, Visualization, Writing – original draft, Writing – review & editing. YL: Formal Analysis, Writing – review & editing. TM: Formal Analysis, Writing – review & editing. YH: Formal Analysis, Writing – review & editing. LZ: Conceptualization, Writing – review & editing.

## Supplementary material

Supplementary Table S1 Physicochemical properties of *TraesTM9SF* genes.

Supplementary Table S2 Protein sequences of *TM9SF* genes from different plant species.

Supplementary Table S3 Coding domains in *TraesTM9SF* sequences.

Supplementary Table S4 Subcellular localization of *TraesTM9SF* genes from different plant species.

Supplementary Table S5 Similarity and identity analysis results of *TraesTM9SF* genes.

Supplementary Data Sheet S1 Functional enrichment of *TraesTM9SF* genes and their TFs in the PPI network in String database.

Supplementary Data Sheet S2 Functional enrichment of *TraesTM9SF* genes from the eggNOG-mapper analysis.

Supplementary Data Sheet S3 List of PDR1-responsive *TraesTM9SF* genes under nitrogen limitation conditions in the profile 14.

Supplementary Data Sheet S4 List of PDR1-responsive *TraesTM9SF* genes under nitrogen limitation conditions in the profile 7.
