# Supplementary material for: Genome-wide analysis of transmembrane 9 superfamily genes in wheat (Triticum aestivum) and their expression in the roots under nitrogen limitation and Bacillus amyloliquefaciens PDR1 treatment conditions
Source: Front Plant Sci. 2024 Jan 8;14:1324974. doi: 10.3389/fpls.2023.1324974 (PMC10800943; doi:10.3389/fpls.2023.1324974)
Supplement: Supplementary Table S1 — Physicochemical properties of TraesTM9SF genes. [file Table_1.docx]

**Additional Table 1 Physicochemical properties of TraesTM9SF genes**

| **Sequence Name** | **Gene Name** | **Chromosome** | **Start** | **End** | **Strand** | **Protein Length (AA)** | **Molecular weight (Dalton)** | **Isoelectric point (pI)** | **GRAVY** | **instaIndex** | **Aliphatic index** | **Theoretical charge** | **Boman index** |
| --- | --- | --- | --- | --- | --- | --- | --- | --- | --- | --- | --- | --- | --- |
| TraesCS2A02G497000.1 | TraesTM9SF-1 | 2A | 727956751 | 727958973 | 1 | 465 | 47720.41 | 9.61 | 0.09 | 29.75 | 65.42 | 20.33 | 1.12 |
| TraesCS7D02G293600.1 | TraesTM9SF-2 | 7D | 364407793 | 364411805 | -1 | 375 | 41039.68 | 9.76 | -0.62 | 21.91 | 42.16 | 27.44 | 2.02 |
| TraesCS6D02G265400.1 | TraesTM9SF-3 | 6D | 375014780 | 375018579 | 1 | 417 | 45304.96 | 10.09 | -0.36 | 30.84 | 51.03 | 29.90 | 1.79 |
| TraesCS5A02G359800.2 | TraesTM9SF-4 | 5A | 561655251 | 561657688 | 1 | 312 | 34423.11 | 10.19 | -0.43 | 25.04 | 48.27 | 27.21 | 1.68 |
| TraesCS5A02G359800.1 | TraesTM9SF-5 | 5A | 561653924 | 561657688 | 1 | 370 | 40942.13 | 10.08 | -0.56 | 25.84 | 45.16 | 30.20 | 1.97 |
| TraesCS5D02G128300.2 | TraesTM9SF-6 | 5D | 200638951 | 200646512 | 1 | 383 | 42126.65 | 10.03 | -0.66 | 32.52 | 43.45 | 29.56 | 2.10 |
| TraesCS5D02G128300.1 | TraesTM9SF-7 | 5D | 200638951 | 200646512 | 1 | 353 | 38603.79 | 10.10 | -0.62 | 32.59 | 44.93 | 30.47 | 1.98 |
| TraesCS7A02G239900.1 | TraesTM9SF-8 | 7A | 214433521 | 214435506 | -1 | 411 | 44357.84 | 10.09 | -0.37 | 32.29 | 48.27 | 28.93 | 1.78 |
| TraesCS4D02G093000.1 | TraesTM9SF-9 | 4D | 68421890 | 68427970 | 1 | 374 | 41189.01 | 9.97 | -0.52 | 25.12 | 48.37 | 29.41 | 1.78 |
| TraesCS7B02G189400.2 | TraesTM9SF-10 | 7B | 323846529 | 323850725 | -1 | 376 | 41267.89 | 9.72 | -0.63 | 25.68 | 42.55 | 26.44 | 2.08 |
| TraesCS7B02G189400.1 | TraesTM9SF-11 | 7B | 323846529 | 323850725 | -1 | 350 | 38770.09 | 9.91 | -0.70 | 27.19 | 39.54 | 29.32 | 2.25 |
| TraesCS2B02G525300.1 | TraesTM9SF-12 | 2B | 719472270 | 719474487 | 1 | 467 | 48189.04 | 9.56 | 0.09 | 32.56 | 64.95 | 19.30 | 1.10 |
| TraesCS7B02G186300.2 | TraesTM9SF-13 | 7B | 307362887 | 307366726 | -1 | 365 | 39959.00 | 9.92 | -0.41 | 22.08 | 49.07 | 26.32 | 1.63 |
| TraesCS7B02G186300.1 | TraesTM9SF-14 | 7B | 307362985 | 307366726 | -1 | 371 | 40762.80 | 10.05 | -0.55 | 17.82 | 47.74 | 29.39 | 1.94 |
| TraesCS7B02G186300.3 | TraesTM9SF-15 | 7B | 307362887 | 307366726 | -1 | 400 | 43864.27 | 9.93 | -0.48 | 20.60 | 48.65 | 29.35 | 1.78 |
| TraesCS6D02G344500.1 | TraesTM9SF-16 | 6D | 444134756 | 444138375 | -1 | 432 | 46376.13 | 9.83 | -0.23 | 33.38 | 54.38 | 25.29 | 1.55 |
| TraesCS6A02G285100.1 | TraesTM9SF-17 | 6A | 516413853 | 516416709 | 1 | 405 | 43892.74 | 10.18 | -0.30 | 26.24 | 52.35 | 33.20 | 1.55 |
| TraesCS7A02G361900.1 | TraesTM9SF-18 | 7A | 536509747 | 536514816 | -1 | 400 | 43731.98 | 9.94 | -0.50 | 20.78 | 48.95 | 29.26 | 1.84 |
| TraesCS7D02G292300.1 | TraesTM9SF-19 | 7D | 355562021 | 355565650 | -1 | 391 | 42835.11 | 9.89 | -0.48 | 18.78 | 48.77 | 28.29 | 1.79 |
| TraesCS6B02G313900.1 | TraesTM9SF-20 | 6B | 561700042 | 561703191 | 1 | 407 | 44126.01 | 10.18 | -0.29 | 26.09 | 53.05 | 33.11 | 1.58 |
| TraesCS4A02G217900.1 | TraesTM9SF-21 | 4A | 518822484 | 518829640 | 1 | 374 | 41201.07 | 9.97 | -0.51 | 23.94 | 49.14 | 29.41 | 1.76 |
| TraesCS7B02G265400.1 | TraesTM9SF-22 | 7B | 487803027 | 487805992 | -1 | 375 | 41430.37 | 9.85 | -0.56 | 21.70 | 47.49 | 25.32 | 1.88 |
| TraesCS5B02G362100.2 | TraesTM9SF-23 | 5B | 541609082 | 541613158 | 1 | 371 | 40431.78 | 10.24 | -0.43 | 22.95 | 48.03 | 33.14 | 1.72 |
| TraesCS5B02G362100.1 | TraesTM9SF-24 | 5B | 541609082 | 541613158 | 1 | 390 | 42599.98 | 10.16 | -0.51 | 23.03 | 45.95 | 32.26 | 1.86 |
| TraesCS4A02G074900.1 | TraesTM9SF-25 | 4A | 74153889 | 74158950 | -1 | 294 | 32537.32 | 9.42 | -0.47 | 30.14 | 42.14 | 18.14 | 1.66 |
| TraesCS5A02G111400.1 | TraesTM9SF-26 | 5A | 214913984 | 214921279 | 1 | 354 | 38628.78 | 10.10 | -0.61 | 32.73 | 45.90 | 30.47 | 1.97 |
| TraesCS5A02G111400.2 | TraesTM9SF-27 | 5A | 214913984 | 214921279 | 1 | 384 | 42151.64 | 10.03 | -0.65 | 32.65 | 44.35 | 29.56 | 2.09 |
| TraesCS6D02G265500.1 | TraesTM9SF-28 | 6D | 375027709 | 375030856 | 1 | 405 | 43967.85 | 10.18 | -0.29 | 25.76 | 53.06 | 33.11 | 1.59 |
| TraesCS4D02G232300.1 | TraesTM9SF-29 | 4D | 392729221 | 392734410 | -1 | 383 | 42066.80 | 9.59 | -0.57 | 27.61 | 44.10 | 25.28 | 1.93 |
| TraesCS2D02G497200.1 | TraesTM9SF-30 | 2D | 593218605 | 593220794 | 1 | 462 | 47688.19 | 9.58 | 0.05 | 29.57 | 65.61 | 19.30 | 1.22 |
| TraesCS7B02G135500.1 | TraesTM9SF-31 | 7B | 165530132 | 165533219 | -1 | 411 | 44378.93 | 10.05 | -0.36 | 32.78 | 47.57 | 28.87 | 1.76 |
| TraesCS7D02G237800.1 | TraesTM9SF-32 | 7D | 202055747 | 202059379 | -1 | 410 | 44275.79 | 10.09 | -0.36 | 32.51 | 47.68 | 28.93 | 1.77 |
| TraesCS6B02G313800.1 | TraesTM9SF-33 | 6B | 561692299 | 561694464 | 1 | 431 | 46764.78 | 10.14 | -0.33 | 29.67 | 50.95 | 33.87 | 1.70 |
| TraesCS7A02G296000.2 | TraesTM9SF-34 | 7A | 392913030 | 392917031 | 1 | 349 | 38628.96 | 9.94 | -0.72 | 28.41 | 38.57 | 30.32 | 2.24 |
| TraesCS7A02G296000.1 | TraesTM9SF-35 | 7A | 392913030 | 392917031 | 1 | 375 | 41010.62 | 9.79 | -0.64 | 25.94 | 41.39 | 28.44 | 2.03 |
| TraesCS6B02G393800.1 | TraesTM9SF-36 | 6B | 668776400 | 668778663 | -1 | 431 | 46317.02 | 9.84 | -0.23 | 34.07 | 54.29 | 25.29 | 1.60 |
| TraesCS7A02G294000.3 | TraesTM9SF-37 | 7A | 377977609 | 377981324 | 1 | 365 | 39987.01 | 9.92 | -0.41 | 22.11 | 49.07 | 26.32 | 1.65 |
| TraesCS7A02G294000.4 | TraesTM9SF-38 | 7A | 377977609 | 377980986 | 1 | 388 | 42599.80 | 9.96 | -0.50 | 18.87 | 49.15 | 28.41 | 1.84 |
| TraesCS7A02G294000.2 | TraesTM9SF-39 | 7A | 377977609 | 377981324 | 1 | 400 | 43892.29 | 9.93 | -0.48 | 20.62 | 48.65 | 29.35 | 1.80 |
| TraesCS7A02G294000.1 | TraesTM9SF-40 | 7A | 377977609 | 377981324 | 1 | 375 | 41291.26 | 10.01 | -0.55 | 23.22 | 46.72 | 30.41 | 1.91 |
| TraesCS4B02G096600.1 | TraesTM9SF-41 | 4B | 100844615 | 100850479 | 1 | 373 | 41101.94 | 9.97 | -0.52 | 23.98 | 48.50 | 29.41 | 1.78 |
| TraesCS6A02G361000.1 | TraesTM9SF-42 | 6A | 591731568 | 591733937 | -1 | 379 | 41068.30 | 9.85 | -0.23 | 34.50 | 55.73 | 22.18 | 1.61 |
| TraesCS7D02G361100.1 | TraesTM9SF-43 | 7D | 463887075 | 463892525 | 1 | 395 | 43433.58 | 9.97 | -0.57 | 21.16 | 47.09 | 29.32 | 1.95 |
| TraesCS5D02G369000.1 | TraesTM9SF-44 | 5D | 444721319 | 444725448 | 1 | 392 | 42868.38 | 10.16 | -0.47 | 23.38 | 47.68 | 32.26 | 1.79 |
| TraesCS6A02G285000.1 | TraesTM9SF-45 | 6A | 516407416 | 516411076 | 1 | 412 | 44712.32 | 10.13 | -0.36 | 31.87 | 50.24 | 30.99 | 1.75 |
| TraesCS5B02G119400.2 | TraesTM9SF-46 | 5B | 211582549 | 211590136 | 1 | 353 | 38591.74 | 10.10 | -0.64 | 32.59 | 44.11 | 30.47 | 2.00 |
| TraesCS5B02G119400.1 | TraesTM9SF-47 | 5B | 211582549 | 211590136 | 1 | 383 | 42114.60 | 10.03 | -0.67 | 32.52 | 42.69 | 29.56 | 2.12 |
